# Identification and expression profile of odorant-binding proteins in the parasitic wasp *Microplitis pallidipes* using PacBio long-read sequencing

**DOI:** 10.1051/parasite/2022053

**Published:** 2022-11-09

**Authors:** Hao Zhang, Jin-Yan Wang, Nian-Feng Wan, Yi-Juan Chen, Xiang-Yun Ji, Jie-Xian Jiang

**Affiliations:** 1 Eco-environmental Protection Institute, Shanghai Academy of Agricultural Sciences, Shanghai Key Laboratory of Protected Horticultural Technology, Shanghai Engineering Research Centre of Low-Carbon Agriculture Shanghai 201403 China; 2 Shanghai Key Laboratory of Chemical Biology, School of Pharmacy, East China University of Science and Technology Shanghai 200237 China

**Keywords:** Biological control, Odorant-binding protein, Parasitic insect, Tissue and temporal expression profile, Transcriptome sequencing

## Abstract

*Microplitis pallidipes* Szépligeti (Hymenoptera: Braconidae) is an important parasitic wasp of second and third-instar noctuid larvae such as the insect pests *Spodoptera exigua*, *Spodoptera litura*, and *Spodoptera frugiperda*. As in other insects, *M. pallidipes* has a chemosensory recognition system that is critical to foraging, mating, oviposition, and other behaviors. Odorant-binding proteins (OBPs) are important to the system, but those of *M. pallidipes* have not been determined. This study used PacBio long-read sequencing to identify 170,980 *M. pallidipes* unigenes and predicted 129,381 proteins. Following retrieval of possible OBP sequences, we removed those that were redundant or non-full-length and eventually cloned five OBP sequences: *MpOBP2*, *MpOBP3*, *MpOBP8*, *MpOBP10*, and *MpPBP* 429, 429, 459, 420, and 429 bp in size, respectively. Each *M. pallidipes* OBP had six conserved cysteine residues. Phylogenetic analysis revealed that the five OBPs were located at different branches of the phylogenetic tree. Additionally, tissue expression profiles indicated that *MpOBP2* and *MpPBP* were mainly expressed in the antennae of male wasps, while *MpOBP3*, *MpOBP8*, and *MpOBP10* were mainly expressed in the antennae of female wasps. *MpOBP3* was also highly expressed in the legs of female wasps. Temporal profiles revealed that the expression of each *M. pallidipes* OBP peaked at different days after emergence to adulthood. In conclusion, we identified five novel odorant-binding proteins of *M. pallidipes* and demonstrated biologically relevant differences in expression patterns.

## Abstract (fr)

*Microplitis pallidipes* Szépligeti (Hymenoptera : Braconidae) est une importante guêpe parasite des larves de noctuelles de deuxième et troisième stades telles que les insectes ravageurs *Spodoptera exigua*, *Spodoptera litura* et *Spodoptera frugiperda*. Comme d’autres insectes, *M. pallidipes* possède un système de reconnaissance chimiosensoriel, essentiel à la recherche de nourriture, à l’accouplement, à la ponte et à d’autres comportements. Les protéines de liaison aux odeurs (PLO) sont importantes pour le système, mais celles de *M. pallidipes* n’ont pas été déterminées. Cette étude a utilisé le séquençage à lecture longue PacBio pour identifier 170 980 unigènes de *M. pallidipes* et prédit 129 381 protéines. Après la récupération des séquences de PLO possibles, nous avons supprimé celles qui étaient redondantes ou pas de pleine longueur et avons finalement cloné cinq séquences de PLO, *MpOBP2*, *MpOBP3*, *MpOBP8*, *MpOBP10* et *MpPBP*, respectivement de taille 429, 429, 459, 420 et 429 pb. Chaque PLO de *M. pallidipes* avait six résidus de cystéine conservés. L’analyse phylogénétique a révélé que les cinq PLO étaient situés à différentes branches de l’arbre phylogénétique. De plus, les profils d’expression tissulaire ont indiqué que *MpOBP2* et *MpPBP* étaient principalement exprimés dans les antennes des guêpes mâles, tandis que *MpOBP3*, *MpOBP8* et *MpOBP10* étaient principalement exprimés dans les antennes des guêpes femelles. *MpOBP3* était également fortement exprimé dans les pattes des guêpes femelles. Les profils temporels ont révélé que l’expression de chaque PLO de *M. pallidipes* culminait à différents jours après l’émergence à l’âge adulte. En conclusion, nous avons identifié cinq nouvelles protéines de liaison aux odeurs de *M. pallidipes* et démontré des différences biologiquement pertinentes dans les profils d’expression.

## Introduction

The chemosensory recognition systems of insects are critical to food-seeking, mating, parasitism, oviposition, and other behaviors [10, 11]. These systems are assisted by odor carriers such as odorant-binding proteins (OBPs), chemosensory proteins (CSPs), odorant receptors (ORs), ionotropic receptors (IRs), and gustatory receptors (GRs), etc. [7, 9]. Mechanistically, OBPs and CSPs transfer odorants to ORs, which recognize and convert chemical signals into electrical signals [21, 33]. The water-soluble OBPs are highly concentrated in the sensillum lymph, functioning to recognize pheromones and chemical odorants in the environment [24, 53]. First identified in antennae of male *Antheraea polyphemus* [46], OBPs have now been widely identified in Lepidoptera, Diptera, Hemiptera, and Coleoptera. The larger protein class is classified into two groups based on functional differences: general odorant-binding proteins (GOBPs, detection of “general” odors such as host plants or food) and pheromone binding proteins (PBPs, involved in the perception of sex pheromones) [23, 34]. Insect OBPs typically have a molecular weight of 15–20 kDa and are characterized by three disulfide bonds formed from six conserved cysteine residues [26, 35].

The number of OBPs varies by insect species. In aphids, OBP count ranges from 9 to 20 [47–48, 50, 59]. *Apis mellifera* has 21 OBPs [13], while *Anopheles gambiae* has 57 [49]. These proteins have also been identified in several parasitic wasp species. For example, *Chouioia cunea* has 25 OBPs [57]; *Dichasma alloeum*, 15 [45]; and *Microplitis mediator*, 20 [36]. Besides this between-species variation, OBPs expression is temporal, sex-, and tissue-specific. For example, in *Locusta migratoria*, *OBP1* and *OBP2* are primarily expressed in female antennae, whereas *OBP6* is mainly expressed in male antennae [25]. *OBP16* of *Helicoverpa armigera* is highly expressed in the wings of the female adults [29]. Two OBPs of *Cybister japonicus* are highly and specifically expressed in male tarsi [41].

The wasp *Microplitis pallidipes* Szépligeti (Hymenoptera: Braconidae), widespread in China, is an important parasite of second and third-instar noctuid larvae, including agricultural pests *Spodoptera exigua*, *Spodoptera litura*, and *Mythimna separata*. Field observations show that *M. pallidipes* parasitizes over 30% of *S. exigua* larvae [52]. Female wasps lay eggs in the body cavity of host caterpillars, and once hatched, *M. pallidipes* larvae obtain nutrients from their hosts, arresting host-caterpillar development at the fourth instar stage. Through the action of OBPs, parasitoids recognize and solubilize volatile hydrophobic odor molecules in the environment to locate hosts for ovipositing [37].

Recent developments in high-throughput transcriptome analysis have contributed greatly to entomology [6, 58]. For example, single-molecule long-read sequencing technology from Pacific Biosciences (PacBio), which can sequence full-length cDNA molecules, has been applied to obtain whole transcriptomes of various species [3, 8, 18]. Methodologically, PacBio transcriptome sequencing can identify alternative isoforms and yields longer reads than Illumina and other second-generation sequencing techniques (SGS) [1]. Third-generation single-molecule sequencing such as the PacBio technology provides novel insights into transcriptome complexity, including complex alternative splicing, full-length splice variants, and alternative polyadenylation.

In general, this is the first transcriptome of the whole body of adults of both sexes of *M. pallidipes*, with the specific goal of identifying OBPs and exploring their spatiotemporal expression profiles by qRT-PCR. We described five OBPs and demonstrated differences in expression patterns across different days after emergence to adulthood, and differences in tissues. Understanding *M. pallidipes* OBPs is potentially useful for developing effective attractants that can elevate the wasp’s effect as a form of biological control on insect pests.

## Materials and methods

### Insects at different days after emergence to adulthood

*Microplitis pallidipes* insects were obtained from the Institute of Eco-environmental Protection, Shanghai Academy of Agricultural Sciences, China. Parasitized *Spodoptera exigua* larvae were fed artificial food in addition to their natural food until they reached the second instar; adult parasitoids were then extracted. Wasps were fed with honey water (10%) and kept under the following environmental conditions: 27 ± 0.5 °C, 85 ± 10% relative humidity, and a 12 h light/dark photoperiod [52].

### RNA extraction and PacBio sequencing

Total RNA used for the PacBio RNA-seq was extracted from one-day-old adult female and male *M. pallidipes* (1:1 sex ratio, ~100 females and 100 males) using Trizol reagent. RNA quality and quantity were determined using gel electrophoresis and an Agilent 2100 Bioanalyzer (Agilent Technologies, Palo Alto, CA, USA), respectively. First-strand cDNA was synthesized from total RNA using PowerScript reverse transcriptase and Oligo-dT primer, following the manufacturer’s protocol (TransGen Biotech, Beijing, China).

### Analysis of PacBio sequencing reads

Raw PacBio polymerase reads with subreads ≥50, and a predicted consensus accuracy ≥0.8 were selected to produce reads of insert (ROIs). These included full-length (FL) and non-full-length (nFL) transcript sequences based on whether 5′/3′ cDNA primers and a poly(A) tail were simultaneously observed. To generate consensus sequences, isoform-level clustering was applied to FL transcripts via the Iso-Seq iterative clustering for error correction (ICE) algorithm. Finally, redundancy in consensus sequences was removed using the CDHIT-EST suite (http://weizhong-lab.ucsd.edu/cdhit_suite/cgi-bin/index.cgi), yielding a full-length transcriptome of *M. pallidipes*.

### Functional annotation of mRNAs

The mRNA sequences were BLAST-searched against the NCBI Non-Redundant (NR; http://www.ncbi.nlm.nih.gov/) and SwissProt (http://www.uniprot.org/) databases. Gene Ontology (GO) (http://www.geneontology.org/) annotations were determined in WEGO (http://wego.genomics.org.cn/), based on the best BLASTX hit from NR. Pathway enrichment analyses were performed using the Kyoto Encyclopedia of Genes and Genomes (KEGG) database and KEGG Automatic Annotation Server (KAAS) (http://www.genome.jp/kegg/kaas/). For both GO and KEGG, threshold *E* values were ≤10^−5^ [32]. Gene functions were predicted and classified using evolutionary genealogy of genes: Non-supervised Orthologous Groups (eggNOG) database (http://eggnog.embl.de).

### Analysis and prediction of TFs, ORFs, and SSRs

Transcription factors (TFs) were identified in Other Eukaryotes TFDB (http://bioinfo.life.hust.edu.cn/AnimalTFDB). Species not included in that database were identified using HMMSEARCH from Protein Family (Pfam). Open reading frames (ORFs) of unigenes were extracted after comparing annotations from NR and SwissProt; ORFs were then translated into protein sequences according to priority. For sequences without NR and SwissProt annotations, ORFs were predicted in Transdecoder. Next, MISA was used to search for simple sequence repeats (SSRs). The minimum number of one-base, two-base, and three-to-six-base repetitions was 10, 6, and 5, respectively. Two SSRs were considered a single compound SSR if the distance between them was less than 100 bp.

### Screening, identification, and sequence analysis of OBPs

Unigene functions were annotated according to the NR database. After searching all annotation results, possible OBPs were classified based on annotation statistics. Sequences were spliced and aligned to obtain possible OBPs, and primers (Table 1) were designed to amplify the full-length sequences using PCR Mix (TransGen, Beijing, China). The thermocycling schedule was: 95 °C for 5 min; followed by 30 cycles at 95 °C for 30 s, 50 °C for 30 s, 72 °C for 45 s; and an additional extension step at 72 °C for 10 min. Amplicons of the expected size were sub-cloned, and three clones were sequenced for each gene. After obtaining full-length OBP sequences, signal peptides were analyzed with SignalP 6.0 (http://www.cbs.dtu.dk/services/SignalP/), and protein domains were analyzed using the Hmmer website (http://www.ebi.ac.uk/Tools/hmmer/) [12]. To conduct a phylogenetic analysis, a total of 61 OBP protein sequences were used from four different insects (21 from *Apis mellifera*, 20 from *Microplitis mediator*, 15 from *Dichasma alloeum*, and five from *Microplitis pallidipes* that we identified). A maximum-likelihood tree based on the Jones–Taylor–Thornton model was constructed in MEGA 7.0, and branch supports were assessed using 1000 bootstrap replicates [22]. The OBP protein sequences were retrieved from GenBank (Supplementary Note 1).

**Table 1:** Primers used for PCR amplification.

Primers	Primer sequences（5′–3′）	Usage of primers
*MpOBP2*exf	ATGAAGTCAATTATTATCTTGGGAGTTTTGCT	Amplify the full-length cDNA gene
*MpOBP2*exr	TTATGTGTACTTGTTGTTGGTCTTGAAG	
*MpOBP3*exf	ATGCGTGGCGTGGTTTTAGCA	
*MpOBP3*exr	TTATTCGTCGTCATGGTGATGGTG	
*MpOBP8*exf	ATGGATTCAAATATAAAATATAT	
*MpOBP8*exr	CTACTGGGTCATTTTCAGAGGC	
*MpOBP10*exf	ATGGCTAAATTTTTGTTGAGC	
*MpOBP10*exr	TTATAGGATTAAATAGGTCTC	
*MpPBP*exf	ATGGTGAGGGTGATACTGAATTACATT	
*MpPBP*exr	TTAAATCATTAACCACATGTCAGGAGAT	
*MpOBP2*-RTexf	CGGAGGCTAAATCGGTACAGAAACG	Real-time PCR
*MpOBP2*-RTexr	CTTGCATCATACGGCCCATGTTTTC	
*MpOBP3*-RTexf	GAAGCAGGAGTCACTAAAG	
*MpOBP3*-RTexr	GTAAATCAGCAGGGAGC	
*MpOBP8*-RTexf	AGTCAAACCAAATATGACCAGCG	
*MpOBP8*-RTexr	TACTGGGTCATTTTCAGAGGCTC	
*MpOBP10*-RTexf	TGCTTATGCTCATTCTGGT	
*MpOBP10*-RTexr	GATGGTGGAG ATAATGCTAA	
*MpPBP*-RTexf	ACATTTTCCTCGGCTTGCTTTTGC	
*MpPBP*-RTexr	TACCCTTCTCTCCTTGAGCCATGTC	
*Mp18S*-RTexf	CGGAGAGGGAGCCTGAGAA	
*Mp18S*-RTexr	CCGGGAGTGGGTAATTTGC	
*Mpβ-actin*-RTexf	TACGCTCTACCTCACGCTATCCTTC	
*Mpβ-actin*-RTexr	CGGCAGTGGTGGTGAAAGAGTATC	

### Real-time quantitative PCR to test stage and tissue OBPs expression profile

To determine the expression profiles of *M. pallidipes* OBPs in different tissues, total RNA used for qRT-PCR analysis was extracted from 20–50 mg of different tissues (antennae, heads from which antennae were removed, thoraxes from which legs and wings were removed, abdomens, wings, and legs) of freshly emerged female and male adult wasps. To determine the expression profile of *M. pallidipes* OBPs at different days after emergence to adulthood, total RNA used for qRT-PCR analysis was extracted from 20–50 mg of whole body of female and male adults at different days (1-day-old adults, 2-day-old adults, 3-day-old adults, 4-day-old adults, and 5-day-old adults). First-strand cDNA was synthesized using the First-Strand cDNA Synthesis Enzyme (TransGen Biotech, Beijing, China). Housekeeping genes were 18S ribosomal RNA gene (MW466574) and *β*-*actin* (MZ570587). TransStart Green qPCR Mix (TransGen, Beijing, China) and appropriate primers (Table 1) were used for amplification. The optimized thermocycling program was 94 °C for 30 s, followed by 45 cycles at 94 °C for 5 s, and 60 °C for 30 s. Data were analyzed on the ABI StepOne instrument (Applied Biosystems, Foster City, CA, USA), and relative gene expression was quantified using the 2^−ΔΔCt^ (cycle thresholds) method [30].

### Statistical analysis

The differences in relative expression of *M. pallidipes* OBPs among different tissues or different days after emergence to adulthood were determined by one-way ANOVA, using statistical package SPSS (Version 22.0, SPSS Inc., Chicago, IL, USA). The difference in relative expression of *M. pallidipes* OBPs between males and females was determined using the *t*-test. Significance was set at *p* < 0.05 in the analysis.

## Results

### Overview of the PacBio sequencing datasets

**Figure 1:**
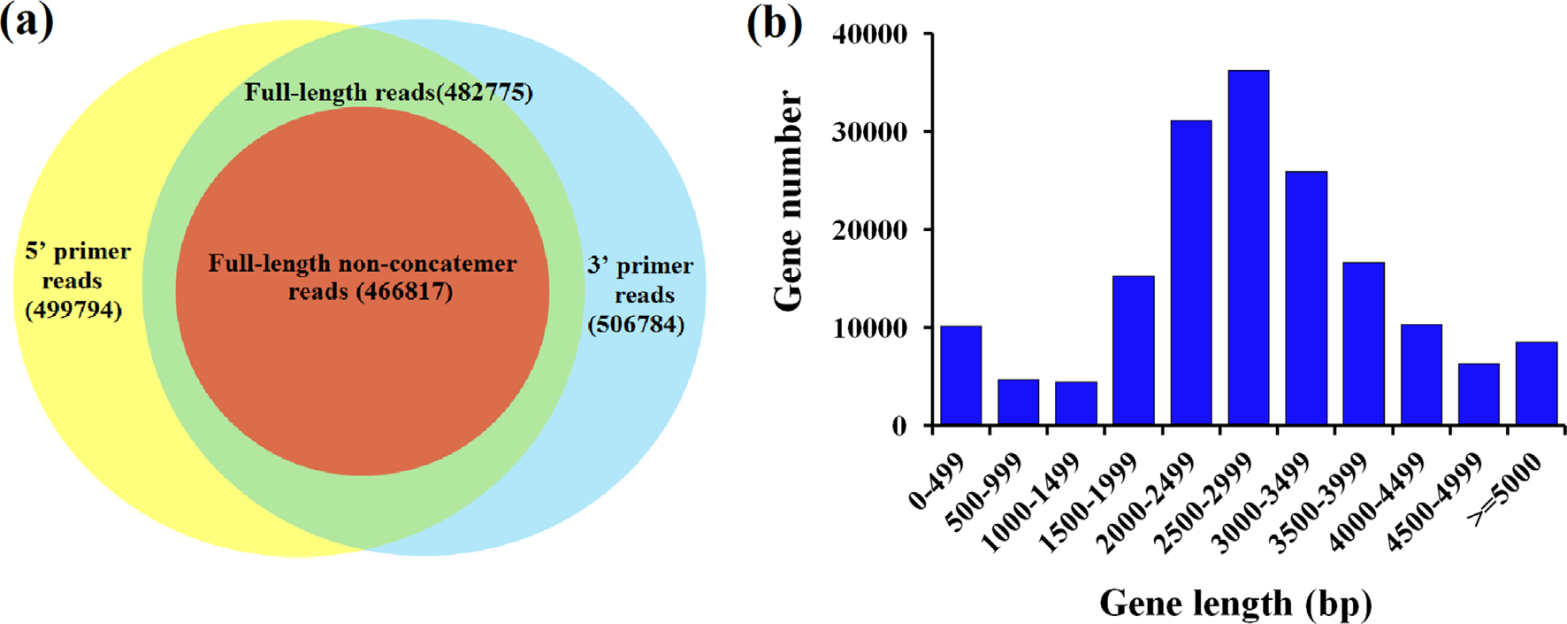
Overview of PacBio sequencing in *Microplitis pallidipes*. (a) Numbers of different types of PacBio reads in *M. pallidipes*. Blue and green circles represent 3′ and 5′ primer reads, respectively while green indicates their intersection. The red circle represents the full-length non-concatemer reads. (b) Length distribution of *M. pallidipes* unigenes obtained using PacBio Iso-Seq. Gene length is on the *x*-axis and number of genes is on the *y*-axis.

PacBio sequencing results showed that the FL transcriptome of *M. pallidipes* contained 520,217 ROIs, including 499,794 5′ primer reads, 506,784 3′ primer reads, 502,379 poly-A reads, 482,775 FL reads, and 37,051 nFL reads. Full-length reads also included 466,817 FL non-concatemer reads with an average length of 2347 bp. After ICE and CD-HIT clustering, we identified 170,980 unigenes with a mean length of 2847.43 bp. The longest sequence length was 19,936 bp, the N50 sequence length was 3184 bp, the N90 sequence length was 2101 bp, and GC content was 34.76%. Length distribution ranged from 500–6000 bp (Fig. 1).

### Functional annotation of the *Microplitis pallidipes* transcriptome

**Figure 2:**
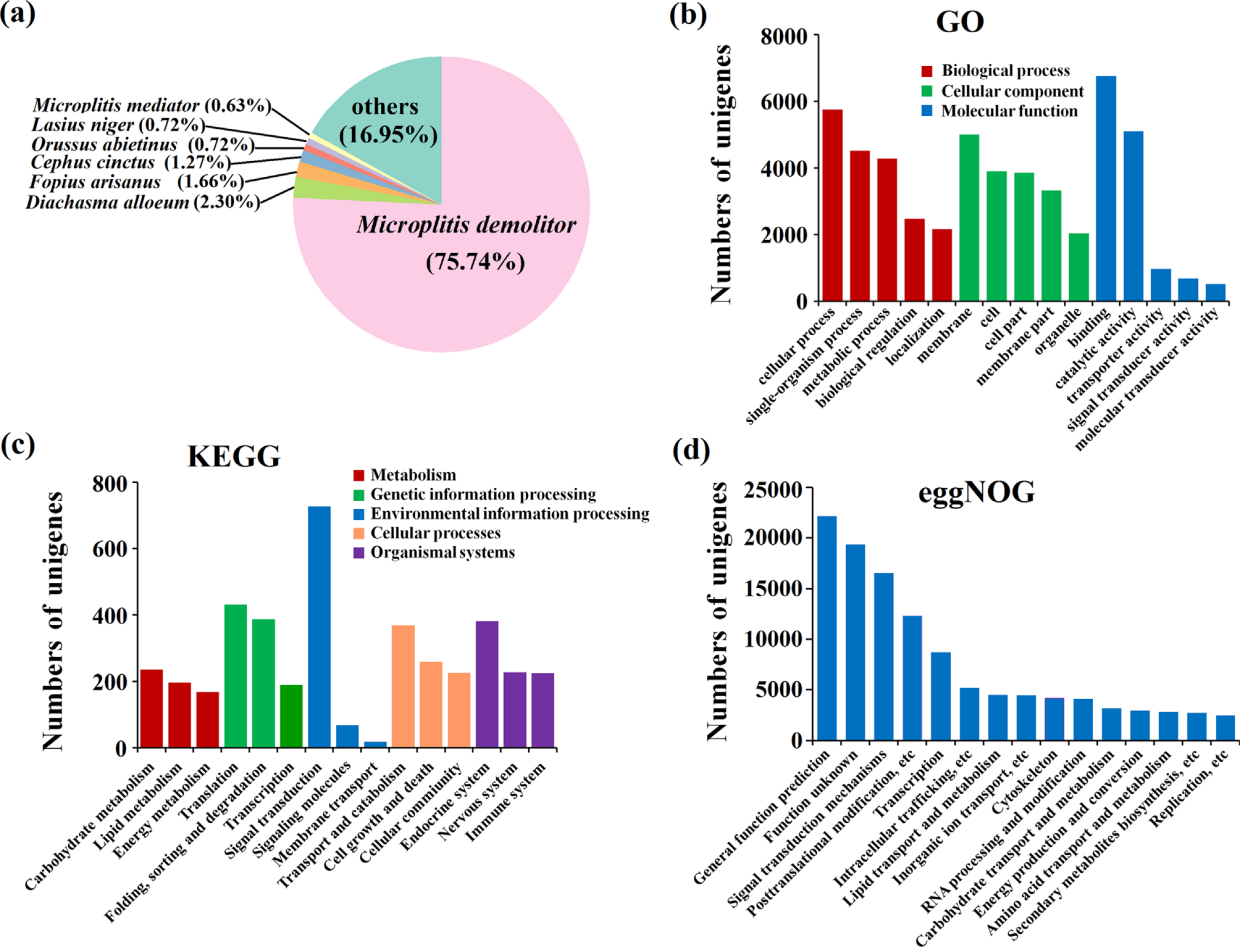
Annotation of transcripts using public databases. (a) Homologous species distribution of *M. pallidipes* annotated using the NR database. (b) GO enrichment analysis: red, biological process; green, cellular component; blue, molecular function. (c) KEGG pathway analysis: red, metabolism; green, genetic information processing; blue, environmental information processing; yellow, cellular processes; purple, organismal systems. (d) eggNOG annotations: eggNOG categories are on the *x*-axis and number of transcripts is on the *y*-axis.

We used five databases (NR, GO, KEGG, eggNOG, and SwissProt) to functionally annotate unigenes. We matched 113,284 unigenes (66.26%), 14,833 unigenes (8.68%), 6282 unigenes (3.67%), 102,405 unigenes (59.89%), 75,277 unigenes (44.03%) to known sequences in the five databases, respectively. We found high-confidence homologs for 2319 unigenes (1.36%) in all five databases. The NR annotation results suggested that 75.74% and 2.3% of the unigenes were matched to *Microplitis demolitor* and *Diachasma alloeum*, respectively (Fig. 2a).

In terms of functional analysis, GO results indicated that most unigenes were enriched in cellular process (biological process), membrane (cellular component), and binding (molecular function) (Fig. 2b). Additionally, KEGG results revealed that most unigenes were involved in pathways related to signal transduction, translation, endocrine system, transport and catabolism, and carbohydrate metabolism (Fig. 2c). Finally, eggNOG results found that most unigenes were predicted to participate in general function (Fig. 2d).

### Analysis and prediction of SSRs, ORFs, and TFs

**Figure 3:**
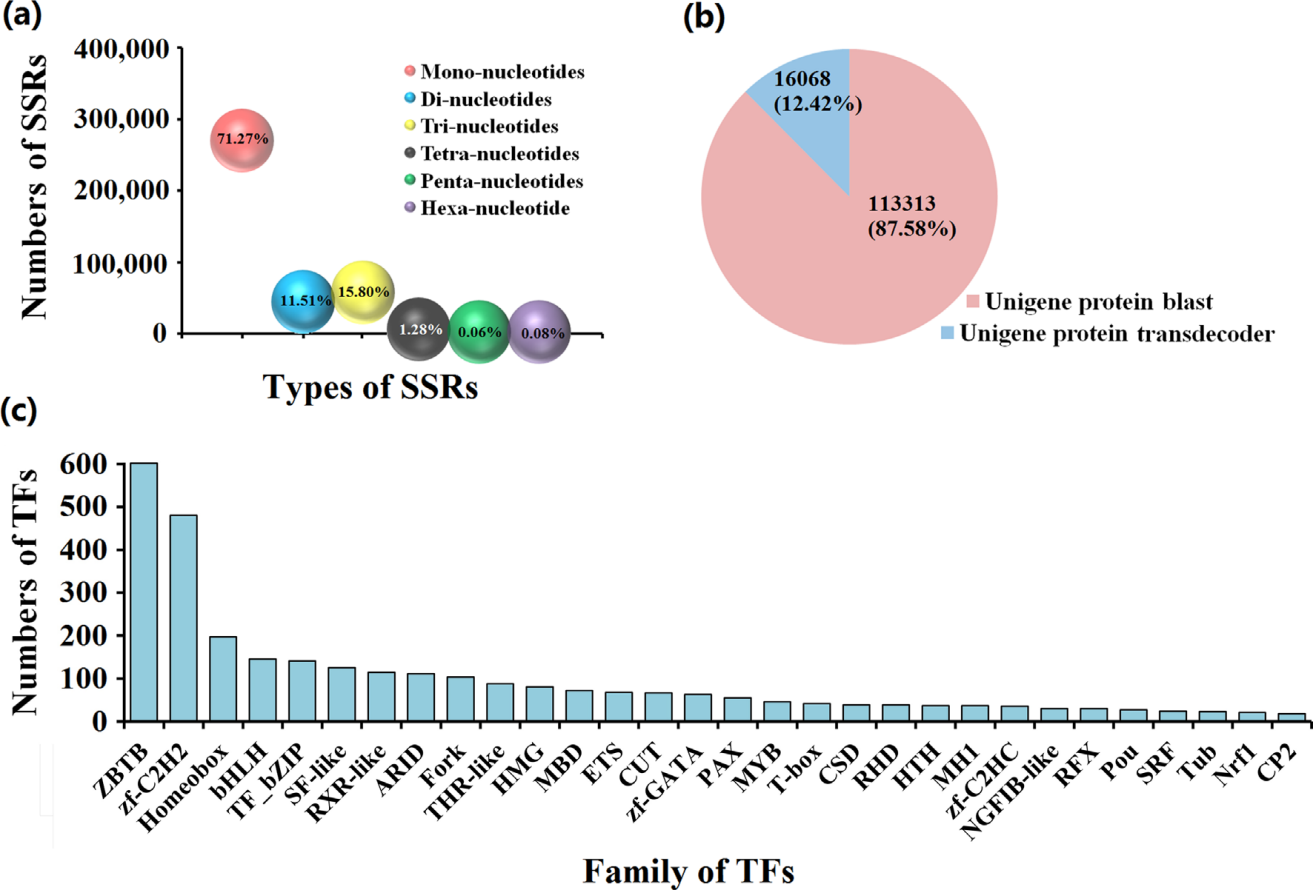
Analysis and prediction of simple sequence repeats, open reading frames, and transcription factors. (a) Simple sequence repeats of *M. pallidipes* transcripts. Number of bases repeated is on the *x*-axis and amount of sequence repeats is on the *y*-axis. (b) Analysis of open reading frames (ORF). Red, ORFs annotated by NR and SwissProt; blue, ORFs predicted using Transdecoder. (c) Transcription factors predicted using SMRT. Transcription-factor family is on the *x*-axis and number of transcription factors is on the *y*-axis.

We identified 378,730 SSRs in the FL transcriptome of *M. pallidipes*. Mononucleotides were the most prevalent (71.27%), followed by trinucleotides (15.80%), dinucleotides (11.51%), tetranucleotides (1.28%), hexanucleotides (0.08%), and pentanucleotides (0.06%) (Fig. 3a). The ORF analysis predicted 129,381 proteins; of these, 113,313 were predicted in NR and SwissProt, while the remaining 16,068 were predicted in Transdecoder (Fig. 3b). The FL transcriptome of *M. pallidipes* contained 3178 TFs. ZTBB (18.94%), Zf-C2H2 (15.10%), Homeobox (6.20%), bHLH (4.56%), and TF_bZIP (4.44%) were the families with the higher number of sequences (Fig. 3c).

### Sequence analysis and phylogenetic analysis of OBPs

**Figure 4:**
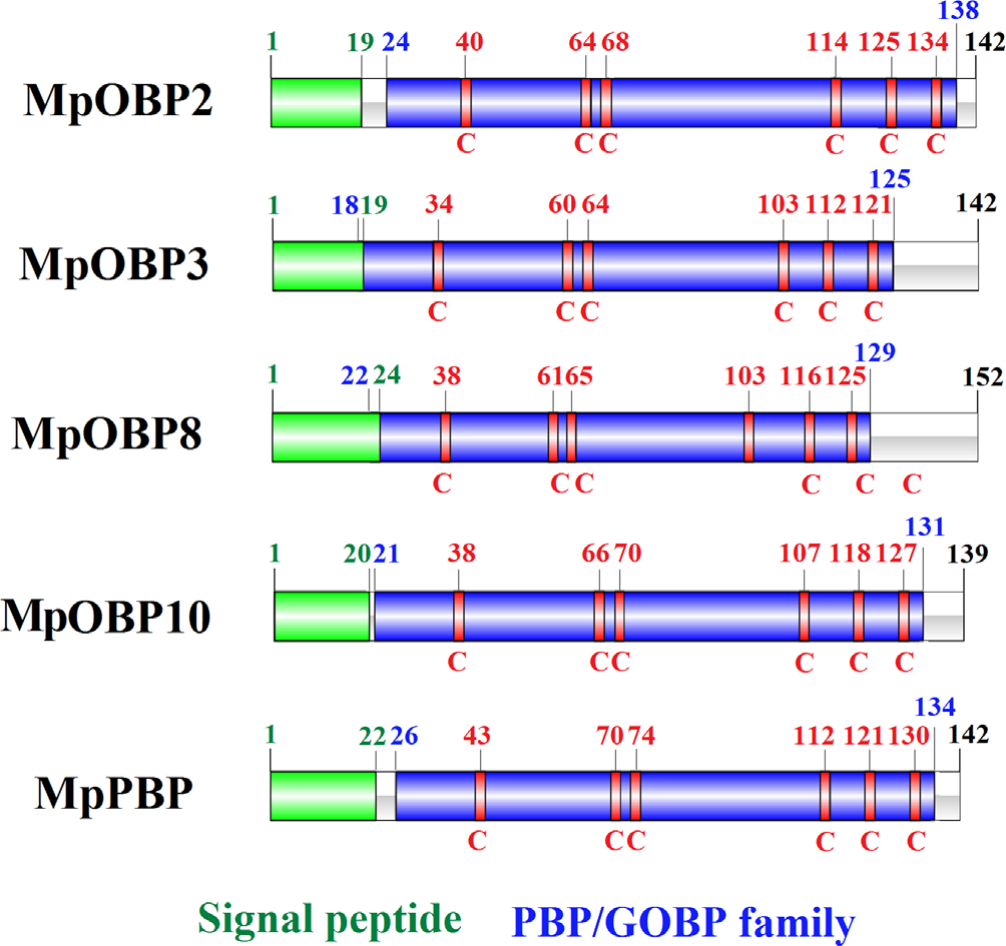
Sequence characteristics analysis of five *M. pallidipes* odorant-binding proteins (OBPs). Green represents signal peptides, blue represents conserved sequences of the PBP/GOBP family, and red represents cysteine. Numbers indicate amino acid position.

From the possible OBP sequences screened, only five full-length sequences were identified after removing redundant, repetitive, and nFL sequences. The five corresponding cDNAs were cloned and sequenced. By comparing the five sequences in NCBI database, it was found that all five sequences had high sequence consistency with other insect OBPs (especially *Microplitis sp.*). These five were *MpOBP2*, *MpOBP3*, *MpOBP8*, *MpOBP10*, and *MpPBP*. they contained 429, 429, 459, 420, and 429 bp, respectively encoding 142, 142, 152, 139, and 142 amino acids (aa). All five *M. pallidipes* OBPs contained signal peptides (*MpOBP2*, *MpOBP3*, *MpOBP8*, *MpOBP10*, and *MpPBP*: 1–19 aa, 1–19 aa, 1–24 aa, 1–20 aa, and 1–22 aa, respectively) and a conserved sequence of the PBP/GOBP family. Finally, each *M. pallidipes* OBP possessed six conserved cysteine residues (Fig. 4).

**Figure 5:**
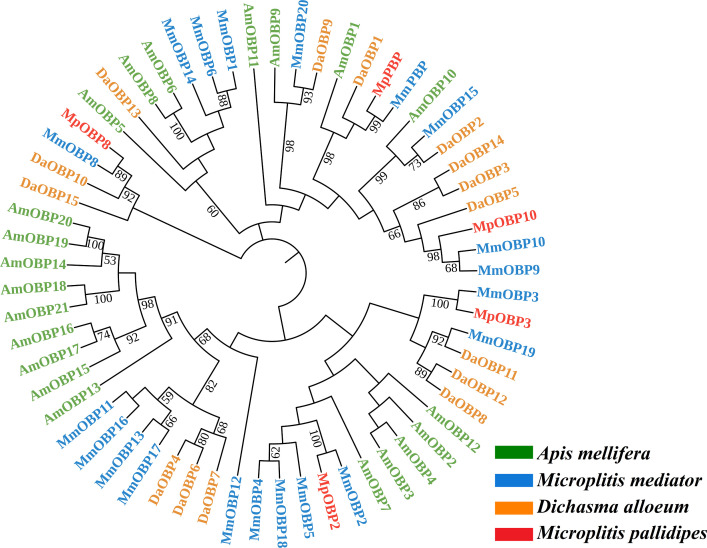
Phylogenetic tree of OBP amino acid sequences reconstructed in MEGA7. Branch lengths indicate evolutionary distances, and numbers represent the tree confidence calculated using bootstrap analysis with 1000 replicates. Different colors of text represent OBPs of different species: red, *Microplitis pallidipes* (Mp); green, *Apis mellifera* (Am); blue, *Microplitis mediator* (Mm); yellow, *Dichasma alloeum* (Da).

Phylogenetic analysis revealed that OBPs generally clustered into three large independent groups. *MpOBP2* and *MpOBP3* were located in a large branch along with their orthologous sequences, and *MpOBP10* and *MpPBP* were located in another large branch. OBP8 was segregated into unique clades with the orthologous sequences. Furthermore, *MpOBP2*, *MpOBP3*, *MpOBP10* and *MpPBP* diverged to different small groups and clustered with their orthologs in other species. MmOBP2 has the closest evolutionary relationship with *MpOBP2* on the phylogenetic tree. This evolutionary relationship also appeared in *MpOBP3*, *MpOBP8*, *MpOBP10*, and *MpPBP* in the two species *M. pallidipes* and *M. mediator* (Fig. 5).

### Tissue and temporal expression profile of OBPs

**Figure 6:**
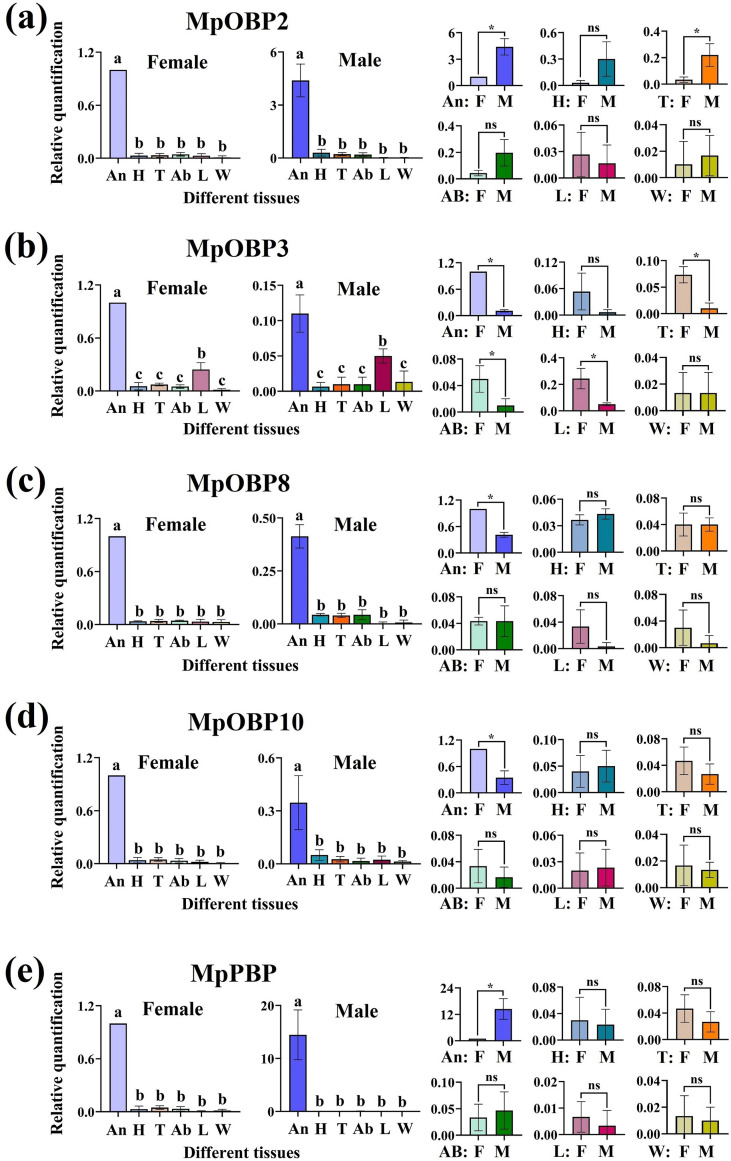
Relative quantification of *M. pallidipes* OBPs in different tissues of female and male wasps. (a–e) *MpOBP2*, *MpOBP3*, *MpOBP8*, *MpOBP10*, and *MpPBP*, respectively. The two charts on the left represent comparisons of OBP expression in different tissues, and the six small charts on the right represent comparisons of female and male OBP expression. Tissue type or sex is on the *x*-axis and relative quantification of *M. pallidipes* OBPs is on the *y*-axis. Bars represent standard deviations. An: antennae, H: heads, T: thoraxes, Ab: abdomens, W: wings, L: legs; F: female, M: male. Different lower-case letters indicate a significant difference (one-way ANOVA, *p* < 0.05). ns means no significant difference and * means a significant difference (*t*-test, *p* < 0.05).

The qPCR results showed that all five *M. pallidipes* OBPs were expressed in the antennae at significantly higher levels surpassing heads, thoraxes, abdomens, wings, and leg tissues for 2.19 to 6197.14 fold-change (*p* < 0.05). There was no significant difference in *MpOBP2* expression level among heads, thoraxes, abdomens, wings, and legs. *MpOBP8*, *MpOBP10*, and *MpPBP* were similar to *MpOBP2*. However, *MpOBP3* expression was significantly higher in legs surpassing heads, thoraxes, abdomens, and wing tissues for 3.32–18.25 fold-change (*p* < 0.05). We also observed several sex differences. *MpOBP2* and *MpPBP* were expressed significantly higher in male antennae than female antennae (ratios of 4.39:1 and 14.46:1, respectively) (*p* < 0.05), whereas *MpOBP8* and *MpOBP10* exhibited the reverse pattern. *MpOBP3* was also highly expressed in the legs of females, in addition to female antennae. Both female antennae and legs typically expressed OBPs at higher levels than male antennae and legs (Fig. 6).

**Figure 7:**
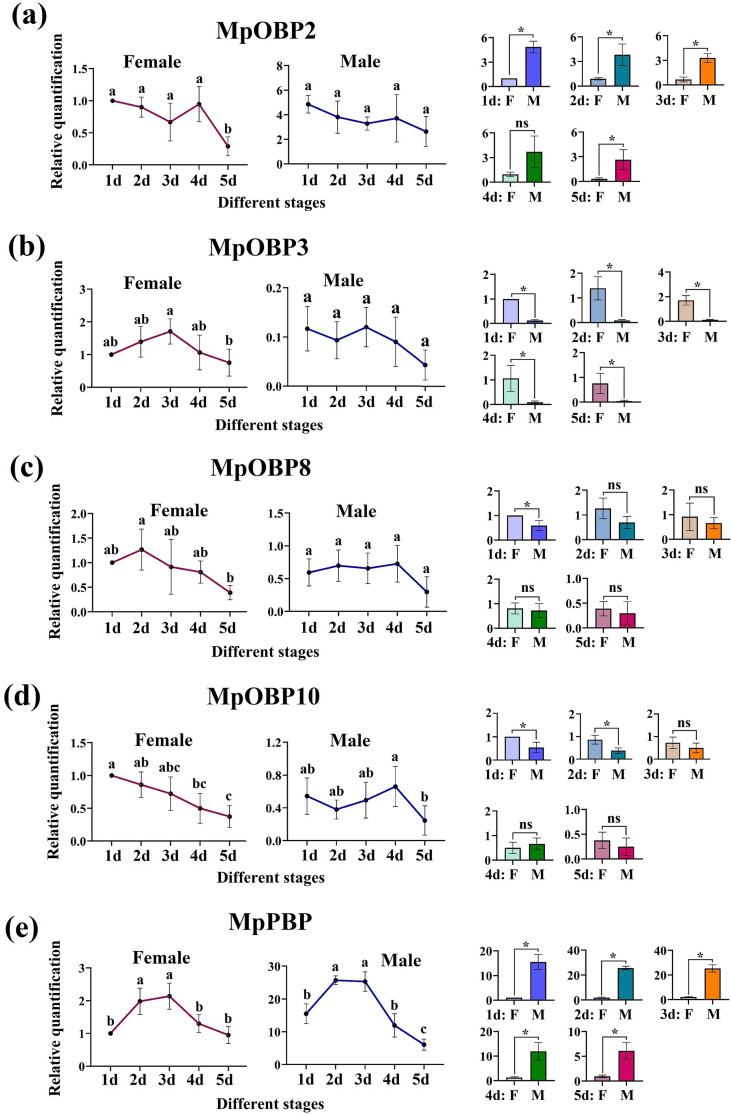
Relative quantification of *M. pallidipes* OBPs at different days after emergence to adulthood in female and male wasps. (a–e) *MpOBP2*, *MpOBP3*, *MpOBP8*, *MpOBP10*, and *MpPBP*, respectively. The two charts on the left represent comparisons of OBP expression at different days after emergence to adulthood, and the five small charts on the right represent comparisons of female and male OBP expression. Day or sex is on the *x*-axis and relative quantification of *M. pallidipes* OBPs is on the *y*-axis. Bars represent standard deviations. 1d–5d: 1-day-old adults, 2-day-old adults, 3-day-old adults, 4-day-old adults, and 5-day-old adults; F: female, M: male. Different lower-case letters indicate a significant difference (one-way ANOVA, *p* < 0.05). ns means no significant difference and * means a significant difference (*t*-test, *p* < 0.05).

Temporal expression profiles also differed across the five OBPs. In female wasps, *MpOBP2* expression of 5-day-old adults was significantly lower than that in 1-day-old, 2-day-old, 3-day-old, and 4-day-old adults (0.29–0.44 fold-change) (*p* < 0.05). *MpOBP3* and *MpPBP* showed the highest expression level in the 3-day-old adults, *MpOBP8* showed the highest expression level in the 2-day-old adults, and *MpOBP10* expression displayed a stepwise decrease from first to fifth day adults. In male wasps, *MpOBP2, MpOBP3,* and *MpOBP8* expression had no significant difference from first to fifth day, respectively. Among the five time points, *MpOBP10* showed the highest expression level in the 4-day-old adults, while *MpPBP* showed the highest expression level in the 2-day-old adults. *MpOBP3* expression was significantly higher in female adults surpassing male adults for 8.57 to 17.38 fold-change from first to fifth day, whereas *MpPBP* exhibited the reverse pattern. *MpOBP2* expression was higher in males surpassing females for 3.92 to 9.08 fold-change from first to fifth day. *MpOBP8* expression was higher in females than in males with the ratio of 1:0.59 in the 1-day-old adults, and there was no significant difference in other adults. *MpOBP10* expression was higher in females than in males in the 1-day-old and 2-day-old adults (Fig. 7).

## Discussion

This study successfully cloned five full-length OBP cDNA from *M. pallidipes* using PacBio long-read sequencing. Our results indicated that *M. pallidipes* had considerably fewer OBPs than many other insects, e.g., *Drosophila melanogaster* with 51 OBPs [17], *Bombyx mori* with 44 [14], *Agrotis ipsilon* with 33 [15], *Encarsia formosa* with 39 [16], *Aulacocentrum confusum* with 11 [28], and *M. mediator* with 20 [36]. The main reason for the relatively low number of *M. pallidipes* OBPs found in this study might be that there was an artifact of some *M. pallidipes* OBPs exhibiting undetectably low or tissue/stage-specific expression. In this study, RNA-Seq analysis was performed on whole body (mixed sexes) to obtain potential OBPs in all tissues. Consequently, the RNA-Seq analysis samples contained fewer specific tissues such as antennae, which meant that the low abundance OBPs specifically expressed in antennae could not be detected. We intend to continue to conduct RNA-Seq analysis on the *M. pallidipes* antennae in future studies to find other OBPs of *M. pallidipes*. In addition, interspecific variation and alternative splicing might also play significant roles in the multiplicity of OBP sequences [19].

The sequence characteristics of *M. pallidipes* OBPs indicated that they belong to the classic OBP family. All five *M. pallidipes* OBPs were predicted to have a conservative PBP-GOBP superfamily domain. Each *M. pallidipes* OBP also contained six conserved cysteine residues, with spacing characteristic of classic OBPs: C1–X_22−27_–C2–X_3_–C3–X_36−45_–C4–X_8−12_–C5–X_8_–C6 (where X is any aa). This pattern differed from those of non-classical OBPs (Dimer OBPs, Plus-C OBPs, Minus-C OBPs, and Atypical OBPs) [14, 19]. Each of the *M. pallidipes* OBP sequences contained a predicted signal peptide typical of secreted proteins, supporting their function in binding and transporting odor molecules [5, 38]. Additionally, the analysis of phylogenetic tree suggested that *MpOBP2* and *MpOBP3* might have a close evolutionary relationship, which was similar with the relationship between *MpOBP10* and *MpPBP*. The close evolutionary relationship between *M. pallidipes* OBPs and *M. mediator* OBPs implied that OBPs are evolutionarily relatively conservative in *Microplitis* sp*.*

Analyzing the tissue-specific pattern of OBPs can provide insight into their biological function. Most insect OBPs are specifically expressed in the antennae, such as the 32 *OBP*s of *Encarsia formosa* [16], nine *OBP*s of *Aulacocentrum confusum* [28], and two *OBP*s of *Macrocentrus cingulum* [2]. Consistent with these prior findings, the five *M. pallidipes* OBPs were primarily expressed in the antennae. However, some OBPs are also expressed in other tissues, such as legs and wings [31, 36]. Examples of high expression in the legs include *OBP2* and *OBP8* of *Aphis glycines* [47], *OBP7* and *OBP16* of *Tropidothorax elegans* [43], and *OBP19* of *M. mediator* [36]. Examples of high expression in the wings include *OBP4* of *Ectropis obliqua* [31], *OBP13* of *Oedaleus infernalis* [54], and *OBP8* of *T. elegans* [43]. Our study found that *MpOBP3* was relatively highly expressed in the legs of female wasps, suggesting involvement in chemo-sensing at the leg level, where many odor receptors related to the olfactory system are distributed [31]. In addition to olfactory perception, OBPs also appear to be involved in other physiological functions, as they are widely expressed in other non-olfactory organs such as the midgut and glands [27, 40, 42]. For example, two *Aedes albopictus* OBPs contribute to transporting hydrophobic ligands in the hemolymph [4]. One *Culex nigripalpus OBP* is associated with nutrient and other small-molecule transport in the intestines [40]. Three OBPs of *Streltzoviella insularis* control semiochemical release in male genitalia [51]. Finally, OBP22 of *Aedes aegypti* is transferred as a pheromone carrier from the male reproductive apparatus to the spermatheca [39].

The expression of OBPs also exhibited sex-specific patterns. *MpOBP2* and *MpPBP* were mainly expressed in female antennae, whereas *MpOBP3*, *MpOBP8*, and *MpOBP10* were mainly expressed in male antennae. These sex-specific patterns are likely related to functional differences. The OBPs expressed specifically on male antennae may be critical for detecting sex pheromones. Alternatively, female-specific OBPs may be important in the detection of general odorants such as host volatiles [20]. Similarly, *OBP14* of *M. mediat*or is also mainly expressed in the female antennae, while *OBP18* of the same species is mostly expressed in male antennae [36].

We observed variable *M. pallidipes* OBP expression across different days after emergence to adulthood. *MpOBP2* and *MpOBP10* expression was highest in 1-day-old wasps, while *MpOBP3* expression was highest in 3-day-old wasps. Furthermore, *MpOBP8* and *MpPBP* expression was highest in 2-day-old wasps. Therefore, we speculated that different OBPs play vital roles at distinct developmental stages. For example, *OBP14* expression in *Adelphocoris lineolatus* fluctuated from its peak in third-instar larvae to its nadir in fourth-instar larvae [44]. Additionally, *OBP11* expression was increased at the late larval and adult stages of *Tribolium castaneum* [55]. OBP1 of *Plutella xylostella* had highest expression level in 1st instar larvae among the different larval stages [56]. These developmental differences might be associated with OBP binding to different odorant compounds, as well as their involvement in a wide range of insect behaviors, including host-searching and mating.

In conclusion, we provide the first mixed transcriptome study of whole body of adults of both sexes of *M. pallidipes* using long-read sequencing and identified five OBP genes. Furthermore, we characterized OBP-gene expression patterns across different tissues and different days after emergence to adulthood using qPCR. These findings provide important insights into OBP function in parasitic wasps and are also applicable to other insects.

## Supplementary material

The supplementary material of this article is available at https://www.parasite-journal.org/10.1051/parasite/2022053/olm. *Note 1*: Protein sequences used in sequence analysis and phylogenetic tree analysis.

## References

[1] Abdel-Ghany SE, Hamilton M, Jacobi JL, Ngam P, Devitt N, Schilkey F, Ben-Hur A, Reddy ASN. 2016. A survey of the sorghum transcriptome using single-molecule long reads. Nature Communications, 7, 11706. 10.1038/ncomms11706 PMC4931028 27339290

[2] Ahmed T, Zhang TT, Wang ZY, He KL, Bai SX. 2017. Molecular cloning, expression profile, odorant affinity, and stability of two odorant-binding proteins in *Macrocentrus cingulum* brischke (Hymenoptera: Braconidae). Archives of Insect Biochemistry and Physiology, 94(2), e21374. 10.1002/arch.21374 28134484

[3] Ali A, Thorgaard GH, Salem M. 2021. PacBio iso-seq improves the rainbow trout genome annotation and identifies alternative splicing associated with economically important phenotypes. Frontiers in Genetics, 12, 683408. 34335690 10.3389/fgene.2021.683408 PMC8321248

[4] Armbruster P, White S, Dzundza J, Crawford J, Zhao XM. 2009. Identification of genes encoding atypical odorant-binding proteins in *Aedes albopictus* (Diptera: Culicidae). Journal of Medical Entomology, 46, 271–280. 19351077 10.1603/033.046.0211

[5] Briand L, Lescop E, Bézirard V, Birlirakis N, Huet JC, Henry C, Guittet E, Pernollet JC. 2001. Isotopic double-labeling of two honeybee odorant-binding proteins secreted by the methylotrophic yeast *Pichia pastoris*. Protein Expression and Purification, 23(1), 167–174. 11570859 10.1006/prep.2001.1478

[6] Caporaso JG, Kuczynski J, Stombaugh J, Bittinger K, Bushman FD, Costello EK, Fierer N, Peña AG, Goodrich JK, Gordon JI, Huttley GA, Kelley ST, Knights D, Koenig JE, Ley RE, Lozupone CA, McDonald D, Muegge BD, Pirrung M, Reeder J, Sevinsky JR, Turnbaugh PJ, Walters WA, Widmann J, Yatsunenko T, Zaneveld J, Knight R. 2010. QIIME allows analysis of high-throughput community sequencing data. Nature Methods, 7(5), 335–336. 20383131 10.1038/nmeth.f.303 PMC3156573

[7] Cheng JF, Yu T, Chen ZJ, Chen S, Chen YP, Gao L, Zhang WH, Jiang B, Bai X, Walker ED, Liu J, Lu YY. 2020. Comparative genomic and transcriptomic analyses of chemosensory genes in the citrus fruit fly *Bactrocera (Tetradacus) minax*. Scientific Reports, 10(1), 18068. 33093485 10.1038/s41598-020-74803-5 PMC7583261

[8] Chin CS, Alexander DH, Marks P, Klammer AA, Drake J, Heiner C, Clum A, Copeland A, Huddleston J, Eichler EE, Turner SW, Korlach J. 2013. Nonhybrid, finished microbial genome assemblies from long-read SMRT sequencing data. Nature Methods, 10(6), 563–569. 23644548 10.1038/nmeth.2474

[9] Du YL, Xu K, Zhao HT, Jiang YS, Li HQ. 2021. Identification and functional characterization of AcerOBP15 from *Apis cerana cerana* (Hymenoptera: Apidae). Apidologie, 52, 668–683.

[10] Eyun S, Soh HY, Posavi M, Munro JB, Hughes DST, Murali SC, Qu JX, Dugan S, Lee SL, Chao H, Dinh H, Han Y, Doddapaneni H, Worley KC, Muzny DM, Park E, Silva JC, Gibbs RA, Richards S, Lee CE. 2017. Evolutionary history of chemosensory-related gene families across the arthropoda. Molecular Biology and Evolution, 34(8), 1838–1862. 28460028 10.1093/molbev/msx147 PMC5850775

[11] Fan J, Francis F, Liu Y, Chen JL, Cheng DF. 2011. An overview of odorant-binding protein functions in insect peripheral olfactory reception. Genetics and Molecular Research, 10(4), 3056–3069. 22180039 10.4238/2011.December.8.2

[12] Finn RD, Clements J, Arndt W, Miller BL, Wheeler TJ, Schreiber F, Bateman A, Eddy SR. 2015. HMMER web server: 2015 update. Nucleic Acids Research, 43, W30–38. 25943547 10.1093/nar/gkv397 PMC4489315

[13] Foret S, Maleszka R. 2006. Function and evolution of a gene family encoding odorant binding-like proteins in a social insect, the honey bee (*Apis mellifera*). Genome research, 16(11), 1404–1413. 17065610 10.1101/gr.5075706 PMC1626642

[14] Gong DP, Zhang HJ, Zhao P, Xia QY, Xiang ZH. 2009. The odorant binding protein gene family from the genome of silkworm *Bombyx mori*. BMC Genomics, 10, 332. 19624863 10.1186/1471-2164-10-332 PMC2722677

[15] Gu SH, Zhou JJ, Wang GR, Zhang YJ, Guo YY. 2013. Sex pheromone recognition and immunolocalization of three pheromone binding proteins in the black cutworm moth *Agrotis ipsilon*. Insect Biochemistry and Molecular Biology, 43, 237–251. 23298680 10.1016/j.ibmb.2012.12.009

[16] He YY, Wang K, Zeng Y, Guo ZJ, Zhang YJ, Wu QJ, Wang SL. 2020. Analysis of the antennal transcriptome and odorant-binding protein expression profiles of the parasitoid wasp *Encarsia formosa*. Genomics, 112(3), 2291–2301. 31899294 10.1016/j.ygeno.2019.12.025

[17] Hekmat-Scafe DS, Scafe CR, McKinney AJ, Tanouye MA. 2002. Genome-wide analysis of the odorant-binding protein gene family in *Drosophila melanogaster*. Genome Research, 12(9), 1357–1369. 12213773 10.1101/gr.239402 PMC186648

[18] Huddleston J, Ranade S, Malig M, Antonacci F, Chaisson M, Hon L, Sudmant PH, Graves TA, Alkan C, Dennis MY, Wilson RK, Turner SW, Korlach J, Eichler EE. 2014. Reconstructing complex regions of genomes using long-read sequencing technology. Genome Research, 24(4), 688–696. 24418700 10.1101/gr.168450.113 PMC3975067

[19] Hull JJ, Perera OP, Snodgrass GL. 2014. Cloning and expression profiling of odorant-binding proteins in the tarnished plant bug, *Lygus lineolaris*. Insect Molecular Biology, 23(1), 78–97. 24224606 10.1111/imb.12064

[20] Jia XJ, Wang HX, Yan ZG, Zhang MZ, Wei CH, Qin XC, Ji WR, Falabella P, Du YL. 2016. Antennal transcriptome and differential expression of olfactory genes in the yellow peach moth, *Conogethes punctiferalis* (Lepidoptera: Crambidae). Scientific Reports, 6(1), 29067. 27364081 10.1038/srep29067 PMC4929561

[21] Jiang XC, Liu S, Jiang XY, Wang ZW, Xiao JJ, Gao Q, Sheng CW, Shi TF, Zeng HR, Yu LS, Cao HQ. 2021. Identification of olfactory genes from the greater wax moth by antennal transcriptome analysis. Frontiers in Physiology, 12, 663040. 34093226 10.3389/fphys.2021.663040 PMC8172125

[22] Kumar S, Stecher G, Tamura K. 2015. Mega 7: molecular evolutionary genetics analysis version 7.0 for bigger datasets. Molecular Biology and Evolution, 33, 1870–1874. 10.1093/molbev/msw054 PMC8210823 27004904

[23] Leal WS. 2013. Odorant reception in insects: roles of receptors, binding proteins, and degrading enzymes. Annual Review of Entomology, 58(1), 373–391. 10.1146/annurev-ento-120811-153635 23020622

[24] Li G, Du J, Li Y, Wu J. 2015. Identification of putative olfactory genes from the oriental fruit moth *Grapholita molesta* via an antennal transcriptome analysis. PLoS One, 10(11), e0142193. 26540284 10.1371/journal.pone.0142193 PMC4635014

[25] Li H, Wang P, Zhang L, Xu X, Cao Z, Zhang L. 2018. Expressions of olfactory proteins in locust olfactory organs and a palp odorant receptor involved in plant aldehydes detection. Frontiers in Physiology, 9, 663. 29915543 10.3389/fphys.2018.00663 PMC5994405

[26] Li MY, Jiang XY, Qi YZ, Huang YJ, Li SG, Liu S. 2020. Identification and expression profiles of 14 odorant-binding protein genes from *Pieris rapae* (Lepidoptera: Pieridae). Journal of Insect Science, 20(5), 1–10. 10.1093/jisesa/ieaa087 PMC7474526 32889524

[27] Li RL, Zhang L, Fang Y, Han B, Li JK. 2013. Proteome and phosphoproteome analysis of honeybee (*Apis mellifera*) venom collected from electrical stimulation and manual extraction of the venom gland. BMC Genomics, 14(1), 766–777. 24199871 10.1186/1471-2164-14-766 PMC3835400

[28] Li YJ, Chen HC, Hong TL, Yan MW, Wang J, Shao ZM, Wu FA, Sheng S, Wang J. 2021. Identification of chemosensory genes by antennal transcriptome analysis and expression profiles of odorant-binding proteins in parasitoid wasp *Aulacocentrum confusum*. Comparative Biochemistry and Physiology Part D Genomics and Proteomics, 40, 100881. 10.1016/j.cbd.2021.100881 34273642

[29] Li ZQ, Zhang S, Zhou SF, Luo JY, Cui JJ. 2017. Tissue expression profiling and ligand-binding properties of HarmOBP16 of the cotton bollworm, *Helicoverpa armigera* (Lepidoptera: Noctuidae). Acta Entomologica Sinica, 60(8), 891–899.

[30] Livak KJ, Schmittgen TD. 2001. Analysis of relative gene expression data using real-time quantitative PCR and the 2(-delta delta c(t)) method. Methods, 25, 402–408. 11846609 10.1006/meth.2001.1262

[31] Ma L, Li ZQ, Lei B, Cai XM, Luo ZX, Zhang YJ, Chen ZM. 2016. Identification and comparative study of chemosensory genes related to host selection by legs transcriptome analysis in the tea geometrid *Ectropis obliqua*. PLoS One, 11(3), e0149591. 26930056 10.1371/journal.pone.0149591 PMC4773006

[32] Moriya Y, Itoh M, Okuda S, Yoshizawa AC, Kanehisa M. 2007. KAAS: an automatic genome annotation and pathway reconstruction server. Nucleic Acids Research, 35, 182–185. 10.1093/nar/gkm321 PMC1933193 17526522

[33] Nishimura O, Brillada C, Yazawa S, Maffei ME, Arimura G. 2012. Transcriptome pyrosequencing of the parasitoid wasp *Cotesia vestalis*: genes involved in the antennal odorant-sensory system. PLoS One, 7(11), e50664. 23226348 10.1371/journal.pone.0050664 PMC3511342

[34] Pelosi P, Iovinella I, Felicioli A, Dani FR. 2014. Soluble proteins of chemical communication: an overview across arthropods. Frontiers in Physiology, 5, 320. 25221516 10.3389/fphys.2014.00320 PMC4145409

[35] Pelosi P, Mastrogiacomo R, Iovinella I, Tuccori E, Persaud KC. 2014. Structure and biotechnological applications of odorant-binding proteins. Applied Microbiology and Biotechnology, 98(1), 61–70. 24265030 10.1007/s00253-013-5383-y

[36] Peng Y, Wang SN, Li KM, Liu JT, Zheng Y, Shan S, Yang YQ, Li RJ, Zhang YJ, Guo YY. 2017. Identification of odorant binding proteins and chemosensory proteins in *Microplitis mediator* as well as functional characterization of chemosensory protein 3. PLoS One, 12(7), e0180775. 28732030 10.1371/journal.pone.0180775 PMC5521769

[37] Ponzio C, Weldegergis BT, Dicke M, Gols R. 2016. Compatible and incompatible pathogen–plant interactions differentially affect plant volatile emissions and the attraction of parasitoid wasps. Functional Ecology, 30, 1779–1789.

[38] Scheuermann EA, Smith DP. 2019. Odor-specific deactivation defects in a *Drosophila* odorant-binding protein mutant. Genetics, 213(3), 897–909. 31492805 10.1534/genetics.119.302629 PMC6827369

[39] Sha L, Picimbon JF, Ji SD, Kan YC, Qiao CL, Zhou JJ, Pelosi P. 2008. Multiple functions of an odorant-binding protein in the mosquito *Aedes aegypti*. Biochemical and Biophysical Research Communications, 372(3), 464–468. 18502197 10.1016/j.bbrc.2008.05.064

[40] Smartt CT, Erickson JS. 2009. Expression of a novel member of the odorant-binding protein gene family in *Culex nigripalpus* (Diptera: Culicidae). Journal of Medical Entomology, 46(6), 1376–1381. 19960683 10.1603/033.046.0617

[41] Song M, Jiang X, Wang XM, Li JD, Zhu F, Tu XB, Zhang ZH, Ban LP. 2016. Male tarsi specific odorant-binding proteins in the diving beetle *Cybister japonicus* sharp. Scientific Reports, 6, 31848. 27545810 10.1038/srep31848 PMC4992826

[42] Song YQ, Song ZY, Dong JF, Lv QH, Chen QX, Sun HZ. 2021. Identification and comparative expression analysis of odorant-binding proteins in the reproductive system and antennae of *Athetis dissimilis*. Scientific Reports, 11(1), 13941. 34230568 10.1038/s41598-021-93423-1 PMC8260659

[43] Song YQ, Sun HZ, Du J. 2018. Identification and tissue distribution of chemosensory protein and odorant binding protein genes in *Tropidothorax elegans* Distant (Hemiptera: Lygaeidae). Scientific Reports, 8(1), 7803. 29773848 10.1038/s41598-018-26137-6 PMC5958050

[44] Sun L, Li Y, Zhang Z, Guo H, Xiao Q, Wang Q, Zhang Y. 2019. Expression patterns and ligand binding characterization of Plus-C odorant-binding protein 14 from *Adelphocoris lineolatus* (Goeze). Biochemical and Molecular Biology, 227, 75–82. 10.1016/j.cbpb.2018.10.001 30292754

[45] Tvedte ES, Walden KKO, McElroy KE, Werren JH, Forbes AA, Hood GR, Logsdon JM, Feder JL, Robertson HM. 2019. Genome of the parasitoid wasp *Diachasma alloeum*, an emerging model for ecological speciation and transitions to asexual reproduction. Genome Biology and Evolution, 11(10), 2767–2773. 31553440 10.1093/gbe/evz205 PMC6781843

[46] Vogt RG, Riddiford LM. 1981. Pheromone binding and inactivation by moth antennae. Nature, 293, 161–163. 18074618 10.1038/293161a0

[47] Wang L, Bi YD, Liu M, Li W, Liu M, Di SF, Yang S, Fan C, Bai L, Lai YC. 2020. Identification and expression profiles analysis of odorant-binding proteins in soybean aphid, *Aphis glycines* (Hemiptera: Aphididae). Insect Science, 27(5), 1019–1030. 31271503 10.1111/1744-7917.12709

[48] Wang Q, Zhou JJ, Liu JT, Huang GZ, Xu WY, Zhang Q, Chen JL, Zhang YJ, Li XC, Gu SH. 2019. Integrative transcriptomic and genomic analysis of odorant binding proteins and chemosensory proteins in aphids. Insect Molecular Biology, 28(1), 1–22. 29888835 10.1111/imb.12513 PMC7380018

[49] Xu PX, Zwiebel LJ, Smith DP. 2003. Identification of a distinct family of genes encoding atypical odorant-binding proteins in the malaria vector mosquito, *Anopheles gambiae*. Insect Molecular Biology, 12, 549–560. 14986916 10.1046/j.1365-2583.2003.00440.x

[50] Xue W, Fan J, Zhang Y, Xu Q, Han Z, Sun J, Chen J. 2016. Identification and expression analysis of candidate odorant-binding protein and chemosensory protein genes by antennal transcriptome of *Sitobion avenae*. PLoS One, 11(8), e0161839. 27561107 10.1371/journal.pone.0161839 PMC4999175

[51] Yang YZ, Li WB, Tao J, Zong SX. 2019. Antennal transcriptome analyses and olfactory protein identification in an important wood-boring moth pest, *Streltzoviella insularis* (Lepidoptera: Cossidae). Scientific Reports, 9(1), 17951. 31784624 10.1038/s41598-019-54455-w PMC6884542

[52] Zhang H, Jiang JX, Chen YJ, Wang JY, Ji XY, Wan NF. 2020. Contribution of a parasitoid species to multiplication and transmission of a multiple nucleopolyhedrovirus in caterpillars. Journal of Applied Entomology, 144(4), 308–314.

[53] Zhang Y, Shen C, Xia D, Wang J, Tang Q. 2019. Characterization of the expression and functions of two odorant-binding proteins of *Sitophilus zeamais* Motschulsky (Coleoptera: Curculionoidea). Insects, 10(11), 409. 10.3390/insects10110409 PMC6920827 31731819

[54] Zhang Y, Tan Y, Zhou XR, Pang BP. 2018. A whole-body transcriptome analysis and expression profiling of odorant binding protein genes in *Oedaleus infernalis*. Comparative Biochemistry and Physiology Part D Genomics and Proteomics, 28, 134–141. 10.1016/j.cbd.2018.08.003 30195212

[55] Zhang YC, Gao SS, Xue S, Zhang KP, Wang JS, Li B. 2020. Odorant-binding proteins contribute to the defense of the red flour beetle, *Tribolium castaneum*, against essential oil of *Artemisia vulgaris*. Frontiers in Physiology, 11, 819. 32982763 10.3389/fphys.2020.00819 PMC7488584

[56] Zhang ZC, Wang MQ, Lu YB, Zhang G. 2009. Molecular characterization and expression pattern of two general odorant binding proteins from the diamondback moth, *Plutella xylostella*. Journal of Chemical Ecology, 35(10), 1188–1196. 19823915 10.1007/s10886-009-9697-2

[57] Zhao Y, Wang FZ, Zhang XY, Zhang S, Guo SL, Zhu GP, Liu Q, Li M. 2016. Transcriptome and expression patterns of chemosensory genes in antennae of the parasitoid wasp *Chouioia cunea*. PLoS One, 11(2), e448159. 10.1371/journal.pone.0148159 PMC4739689 26841106

[58] Zhou CX, Min SF, Tang YL, Wang MQ. 2015. Analysis of antennal transcriptome and odorant binding protein expression profiles of the recently identified parasitoid wasp, *Sclerodermus sp*. Comparative Biochemistry and Physiology Part D Genomics and Proteomics, 16, 10–19. 10.1016/j.cbd.2015.06.003 26164593

[59] Zhou JJ, Vieira FG, He XL, Smadja C, Liu R, Rozas J, Field LM. 2010. Genome annotation and comparative analyses of the odorant-binding proteins and chemosensory proteins in the pea aphid *Acyrthosiphon pisum*. Insect Molecular Biology, 19(S2), 113–122. 10.1111/j.1365-2583.2009.00919.x 20482644

