# Supplementary material for: Identification and expression profile of odorant-binding proteins in the parasitic wasp Microplitis pallidipes using PacBio long-read sequencing
Source: Parasite. 2022 Nov 9;29:53. doi: 10.1051/parasite/2022053 (PMC9645227; doi:10.1051/parasite/2022053)
Supplement: Supplementary file 1 — Note 1: Protein sequences used in sequence analysis and phylogenetic tree analysis. [file parasite-29-53-s1.pdf]

1. The protein sequences used in sequence analysis and phylogenetic tree analysis.

Microplitis pallidipes**:** MpOBP2, UUL48238.1; MpOBP3, UUL48239.1; MpOBP8, AYD59728.1; MpOBP10, UUL48240.1; MpPBP, UUL48241.1. ***Apis mellifera***: AmOBP1, NP_001011590.1; AmOBP2, NP_001011591.1; AmOBP3, NP_001035311.1; AmOBP4, NP_001011589.1; AmOBP5, NP_001011588.1; AmOBP6, NP_001011593.1; AmOBP7, NP_001035310.1; AmOBP8, NP_001164515.1; AmOBP9, NP_001035315.1; AmOBP10, NP_001035294.1; AmOBP11, NP_001035316.1; AmOBP12, NP_001035319.1; AmOBP13, NP_001035314.1; AmOBP14, NP_001035313.1; AmOBP15, NP_001035298.1; AmOBP16, NP_001035295.1; AmOBP17, NP_001035297.1; AmOBP18, NP_001035317.1; AmOBP19, NP_001035299.1; AmOBP20, NP_001035312.1; AmOBP21, NP_001035296.1. ***Diachasma alloeum***: DaOBP1, THK33212.1; DaOBP2, THK33147.1; DaOBP3, THK33146.1; DaOBP4, THK33045.1; DaOBP5, THK32955.1; DaOBP6, THK33257.1; DaOBP7, THK33256.1; DaOBP8, THK33255.1; DaOBP9, THK32847.1; DaOBP10, THK32846.1; DaOBP11, THK33067.1; DaOBP12, THK33066.1; DaOBP13, THK33083.1; DaOBP14, THK33119.1; DaOBP15, THK32894.1. ***Microplitis mediator***: MmOBP1, ABM05968.2; MmOBP2, ABM05969.1; MmOBP3, ABM05970.1; MmOBP4, ABM05971.2; MmOBP5, ABM05972.1; MmOBP6, ABO15559.1; MmPBP, ABM05973.2; MmOBP8, AEF14409.1; MmOBP9, https://doi.org/10.1371/journal.pone.0180775.g001; MmOBP10, AEO27860.1; MmOBP11, ANT46040.1; MmOBP12, ANT46041.1; MmOBP13, ANT46042.1; MmOBP14, ANT46043.1; MmOBP15, ANT46044.1; MmOBP16, ANT46045.1; MmOBP17, ANT46046.1; MmOBP18, ANT46047.1; MmOBP19, ANT46048.1; MmOBP20, ANT46049.1.

;
